# Near Field 3-D Millimeter-Wave SAR Image Enhancement and Detection with Application of Antenna Pattern Compensation

**DOI:** 10.3390/s22124509

**Published:** 2022-06-14

**Authors:** Shaoqiu Song, Jie Lu, Shiqi Xing, Sinong Quan, Junpeng Wang, Yongzhen Li, Jing Lian

**Affiliations:** College of Electronic Science and Technology, National University of Defense Technology, Changsha 410073, China; songshaoqiussq@163.com (S.S.); jielu19@outlook.com (J.L.); qsnong@hotmail.com (S.Q.); jpwang1998@163.com (J.W.); e0061@sina.com (Y.L.); lianjing20@nudt.edu.cn (J.L.)

**Keywords:** SAR, near-field, mmWave, high-fidelity, image reconstruction focusing, kappa coefficients

## Abstract

In this paper, a novel near-field high-resolution image focusing technique is proposed. With the emergence of Millimeter-wave (mmWave) devices, near-field synthetic aperture radar (SAR) imaging is widely used in automotive-mounted SAR imaging, UAV imaging, concealed threat detection, etc. Current research is mainly confined to the laboratory environment, thus ignoring the adverse effects of the non-ideal experimental environment on imaging and subsequent detection in real scenarios. To address this problem, we propose an optimized Back-Projection Algorithm (BPA) that considers the loss path of signal propagation among space by converting the amplitude factor in the echo model into a beam-weighting. The proposed algorithm is an image focusing algorithm for arbitrary and irregular arrays, and effectively mitigates sparse array imaging ghosts. We apply the 3DRIED dataset to construct image datasets for target detection, comparing the kappa coefficients of the proposed scheme with those obtained from classic BPA and Range Migration Algorithm (RMA) with amplitude loss compensation. The results show that the proposed algorithm attains a high-fidelity image reconstruction focus.

## 1. Introduction

With the increased popularity of low-cost commercial Millimeter-wave (mmWave) radar sensors, high-resolution 3-D mmWave imaging systems have recently attracted a wide range of attention [1]. Moreover, it now plays an essential role in many applications, including gesture recognition [2], medical imaging [3,4], automotive-mounted SAR imaging [5,6], UAV imaging, non-destructive testing, and concealed threat detection [7], etc. The success behind the works is partially due to the millimeter radar sensors, which have smaller sizes, higher integration levels, and broader bandwidth [2]. mmWave can penetrate objects such as various composites, wood, clothing, etc. A signal at the mmWave is also non-ionizing and not considered a dangerous radiation source [4]. Although the radar hardware is constantly updated, it is still a considerable challenge to obtain millimeter resolution images. An effective way to acquire high-resolution data is through SAR. The mmWave radar continuously obtains the synthetic aperture by adopting a planar scanning pattern in space at the expense of time complexity to achieve high-resolution imaging. In terms of imaging algorithms, the Back-Projection Algorithm (BPA) [8] and Range Migration Algorithm (RMA) [9,10] are regarded as the most classic algorithms in near-field radar imaging [11,12]. BPA can be used in any array configuration, which offers a time-domain solution to estimate the target scattering coefficient by traversing all imaging grid points to calibrate echo data. High computational loads are necessary for larger data volumes, severely reducing computational efficiency. The RMA using Fourier transform is the most efficient and widely used method in SAR imaging, which is a frequency-domain fast imaging algorithm calculated by converting the echo signal to wavenumber-domain for phase correction. However, the approach is limited as it is only applicable to regular spatial intervals. These algorithms perform image reconstruction from different domains by inverting radar echoes into target scattering factors [13,14,15]. Most imaging algorithms research is based on these two models for optimization. Near field imaging, fast imaging [15], sparse imaging [16,17], MIMO array design [18], and optimization are frequent research topics [19,20,21]. In recent years, the compression-sensing algorithm(CSA) has been a novel technique [22,23]. Since uniform arrays are costly, and the incredibly massive number of arrays often result in complex data processing, the CSA could take full advantage of the sparse characteristics of the target. The CSA requires an extremely high signal-to-noise ratio and also requires extremely accurate physical models to achieve good accuracy at sub-Nyquist sampling. Sun et al. [23] researched the NUFFT algorithm to accelerate CS radar imaging, two fast Gaussian lattice point-based NUFFT methods to expedite the CS process for solving computationally intensive problems in large-scale and real-time radar imaging. Kajbaf H. et al. [24] investigated 3-D microwave imaging compression-sensing processing and mainly discussed the inhomogeneous grid and optimal path sparse sampling. Compared with the SISO-SAR imaging algorithm, separating the transceiver array elements with the MIMO-SAR [25,26,27] should consider the different trajectories of the reflected and transmitted electromagnetic waves, instead of directly applying the equivalent phase model, which makes the signal processing more complicated [28,29,30,31,32]. Yanik, M.E. [12] from the University of Texas pioneered a near-field imaging system with commercially available low-cost industrial-grade mmWave MIMO radar sensors, and explored the concept of virtual antenna array in near-field MIMO SAR, incorporating a multistatic-to-monostatic correction factor to improve image formation. Wang J [31] proposed an approach utilizing the NUFFT algorithm to estimate the signal spectra over a rectilinear grid in the frequency-wavenumber (i.e., f-k) domain from the spatial signals measured by a 2-D MIMO array. Finally, Smith, J.W. [32] introduced the efficient 3-D near-field MIMO-SAR imaging for irregular scanning geometries.

However, the existing literature has not thoroughly discussed the suppression of the imaging clutter or ghost due to the non-ideal electromagnetic environment or sparse arrays during the imaging process [33]. Although practical progress has been made toward the current imaging optimization algorithms, most of them are performed under darkroom conditions and are not generally applicable to security detection scenarios [34,35,36,37,38,39,40,41,42,43].

In this article, from the essence of imaging, we propose a novel image reconstruction focusing technique for the efficient near-field imaging in non-ideal electromagnetic environments or sparse arrays, such as those present in security screening imaging, concealed object imaging, automotive, and UAV SAR. We examine the signal models and system for SAR and develop a method to enhance the imaging quality in terms of the radar equation and the antenna beam. This technique extends the BPA to optimize coherent accumulation. The measured results validate the robustness of the proposed algorithm in a complex environment or sparse arrays, and the analysis in the subsequent sections provides an intensive evaluation of the difference in parameter metrics between the technique and traditional imaging algorithms. Finally, we use the 3DRIED [35] to construct the image datasets under these three algorithms for practical inspection and validation. The proposed method indicates a higher fidelity focus comparable to the traditional planar RMA and BPA, even under non-ideal electromagnetic environments.

Within the article, Section 2 introduces the system models, including the signal model, the RMA with Amplitude Loss Compensation and BPA, a novel enhancement technique to planar BPA, and YOLO detection technique. Section 3 discusses imaging results and parameter metrics presented. Section 4 constructs the image datasets to perform target detection recognition to demonstrate the superiority of the proposed algorithm. Conclusively, Section 5 summarizes the whole paper and then concludes.

## 2. Relevant Research Theories

### 2.1. Signal Model

**Figure 1:**
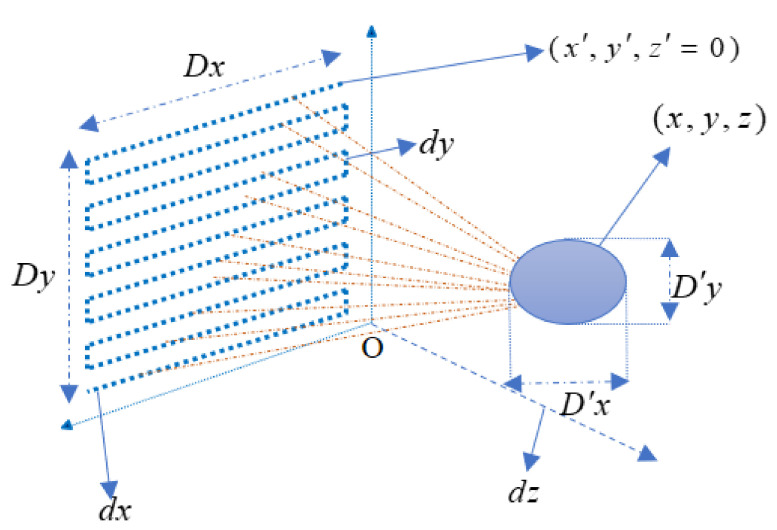
The geometry of the SISO-SAR imaging configuration, where a planar aperture is synthesized by Z−shaped mechanical moving.

This paper uses the FMCW radar system to evaluate the performance metrics of multiple imaging algorithms in complex environments, with the specific radar measurement scenario as shown in Figure 1. The traditional signal model for FMCW is well described in the literature. Here, the FMCW radar emits a linear frequency modulated signal called chirp

(1) $$m(t)=e^{j2\pi (f_{c}t+0.5Kt^{2})}\;0\leq t\leq T_{c}$$

where $f_{c}$ is the instantaneous carrier frequency at the time t=0, $K=B/T_{c}$ is the chirp of the frequency slope. B is the signal of sweep band and $T_{c}$ is the duration during the fast time.

The signal s(t) is the radar transmitted signal, reflected by the scattering point. The radar received antenna accepts the returned echo signal as a time delay of the transmitted signal. It is known as de-chirping and leads to a complex intermediate frequency signal [37]

(2) $$\overset{⌢}{m}(t)=\sigma \frac{1}{4\pi R_{t}R_{r}}e^{j2\pi (f_{c}(t-\tau )+0.5K{\left(t-\tau \right)}^{2})}$$

where τ is the pulse round trip time delay, $R_{t}$ and $R_{r}$ is the path of electromagnetic wave emission and reflection which can be described as

(3) $$\begin{array}{l} R_{t}=\sqrt{{\left(x-x_{t}\right)}^{2}+{\left(y-y_{t}\right)}^{2}+(z-z_{t})}\\ R_{r}=\sqrt{{\left(x-x_{r}\right)}^{2}+{\left(y-y_{r}\right)}^{2}+(z-z_{r})} \end{array}$$

where $\tau =(R_{t}+R_{r})/c$, as the transmitted and received antennas are not located at the same point. This is equated to the co-location of the received and transmitted antennas to facilitate the subsequent signal processing. Unlike the direct EPC model for far fields, this approach of near-field approximation has been discussed and researched in the literature [32].

(4) $$2R=R_{t}+R_{r}\approx 2R^{\prime }+\frac{{\left(d_{x}^{l}\right)}^{2}+{\left(d_{y}^{l}\right)}^{2}}{4z_{0}}\;R^{\prime }=\sqrt{{\left(x-x^{\prime }\right)}^{2}+{\left(y-y^{\prime }\right)}^{2}+{\left(z-z^{\prime }\right)}^{2}}$$

where R is the range between the virtual array element and the target scattering point, $d_{x}^{l}$ and $d_{y}^{l}$ are the horizontal and vertical distances between the transmitted and received antennas.

Therefore, the received signal $\overset{⌢}{m}(t)$ is mixed with m(t) to generate a fundamental IF signal, which can be expressed as

(5) $$s(t)=\frac{\sigma }{8\pi R^{2}}e^{j2\pi (K\tau t+f_{c}\tau -0.5K\tau^{2})}$$

The first phase term of (5) is the beat frequency representing range information, and the second phase term means the Doppler phase, which is the critical factor in SAR imaging. The last phase term is known as the residual video phase (RVP) to be ignored [37]. Finally, the signal model can be represented as

(6) $$s(k)=A\sigma \frac{e^{j2kR}}{R^{2}}\frac{2\pi f_{c}}{c}\leq k\leq \frac{2\pi f}{c}$$

where f(x,y) is the scattering coefficient function and k=2πf/c, $f=f_{c}+Kt$. A is the constant term. f indicates the sampling frequency at each sampling point on the signal carrier frequency and k is the wavenumber. Expanding the signal model in (6), we can obtain

(7) $$s(x^{\prime },y^{\prime },k)=\iint \frac{f(x,y,z)}{R^{2}}e^{j2kR}dxdydz$$

The mmWave radar scans a 2-D array by Z shaped mechanical moving to obtain the 3D raw echo data. Therefore, large bandwidth and high resolution in azimuth and height are obtained. To ensure no ghost image, the horizontal and vertical synthetic aperture with spatial sampling interval dx and dy should be satisfied with Nyquist criterion. λ is the wavelength. For the worst case situation that the target is very close to the radar, the aperture spatial interval should be less than λ/4.Traditionally, the aperture spatial interval between λ/4 and λ/2 is the “industry standard” [2]. Furthermore, the resolution of the range direction is determined by the system bandwidth. Specifically, the lower the bandwidth, the larger the range direction resolution for the radar to distinguish two targets. For the 3-D near-field radar imaging system, the spatial resolution [40] along each dimension can be given by

(8) $$\begin{array}{l} \delta x\approx \frac{\lambda \sqrt{{\left[\left(Dx+D^{\prime }x\right)/4\right]}^{2}+{\left(z-z^{\prime }\right)}^{2}/4}}{Dx+D^{\prime }x}dx\leq \delta x\\ \delta y\approx \frac{\lambda \sqrt{{\left[\left(Dy+D^{\prime }y\right)/4\right]}^{2}+{\left(z-z^{\prime }\right)}^{2}/4}}{Dy+D^{\prime }y}\;dy\leq \delta y\\ \delta z\approx \frac{c}{2(f-f_{c})}\approx \frac{c}{2B} \end{array}$$

where Dx and Dy are the width of the aperture along the $x^{\prime }$ and $y^{\prime }$ direction of a coordinate system, respectively, $D^{\prime }x$ and $D^{\prime }y$ are the width of the aperture along the x and y direction of the target. Furthermore, the spatial resolution [7] along azimuth and height dimensions can be simply expressed as

(9) $$\begin{array}{l} \delta x\approx \frac{\lambda z_{0}}{2Dx}\\ \delta y\approx \frac{\lambda z_{0}}{2Dy} \end{array}$$

### 2.2. Imaging Algorithm

#### 2.2.1. Range Migration Algorithm with Amplitude Loss Compensation

The Fourier-based algorithm in the subsequent analysis is known as the range migration algorithm or ω−k algorithm, which has been widely discussed in more detail elsewhere [2,32]. The amplitude factor and amplitude loss are usually ignored in RMA. Here, we take the amplitude loss into account for a stationary target whose distance $Z_{0}=\left|z-z^{\prime }\right|$ is constant. The imaging reconstruction will have the phase direction and the preserved first order ${\left(Z_{0}R\right)}^{-1}$ to obtain. This amplitude approximation method has been mentioned in the literature [41]. As a result, we can yield

(10) $$s(x^{\prime },y^{\prime },k)=∭f(x,y,z)\frac{e^{j2kR}}{R}dxdydz$$

The next derivation is based on the fluctuation equation for spherical waves equivalent to plane waves [30,43]. We can yield

(11) $$s(x^{\prime },y^{\prime },k)=∭\frac{j}{2\pi }f(x,y,z)\iint \frac{e^{j[k_{x}(x^{\prime }-x)+k_{y}(y^{\prime }-y)+k_{z}(z-z^{\prime })]}}{k_{z}}dk_{x}dk_{y}dxdydz$$

where $k_{x}$, $k_{y}$, $k_{z}$ are the azimuthal-height-range direction of the wavenumber k, and it is vital to note that $4k^{2}=k_{x}{}^{2}+k_{y}{}^{2}+k_{z}{}^{2},k_{x}{}^{2}+k_{y}{}^{2}\leq 4k^{2}$ is the critical constraint for image reconstruction. Leveraging conjugate symmetry of the spherical wavefront, we can obtain

(12) $$s^{*}(x^{\prime },y^{\prime },k)=\frac{j}{2\pi }\iint [∭f(x,y,z)e^{-j(k_{x}x+k_{y}y+k_{z}z)}dxdydz]\frac{e^{jk_{z}z^{\prime }}}{k_{z}}e^{j(k_{x}x^{\prime }+k_{y}y^{\prime })}dk_{x}dk_{y}$$

where ${\left(\cdot \right)}^{*}$ is the complex conjugate operation. In the Fourier transform operation $FFT_{2D}\;\mathrm{and}\;IFFT_{2D}$, the above difference between the primed and unprimed coordinate systems is dropped, as they are coincident. Hence, Equation (9) becomes

(13) $$\begin{array}{l} s^{*}(x,y,k)\\ =\frac{j}{2\pi }\iint F(k_{x},k_{y},k_{z})\frac{e^{jk_{z}z^{\prime }}}{k_{z}}e^{j(k_{x}x+k_{y}y)}dk_{x}dk_{y}=\frac{j}{2\pi }IFFT_{2D}\left[F(k_{x},k_{y},k_{z})\frac{e^{jk_{z}z^{\prime }}}{k_{z}}\right] \end{array}$$

The time-domain convolution corresponds to the frequency-domain product. Removing constant terms and combining Equations (13) can be rewritten as

(14) $$\begin{array}{l} s^{*}(k_{x},k_{y},k)=FFT_{2D}\left[f(x,y,k_{z})⊗h(x,y)\right]=F(k_{x},k_{y},k_{z})H(k_{x},k_{y})\\ H(k_{x},k_{y})=\frac{e^{jk_{z}z^{\prime }}}{k_{z}} \end{array}$$

Therefore, the interpolation is used to resample the data cube to uniformly spaced positions in $k_{z}$, and the 3-D image reconstruction can be carried out as [44]

(15) $$f(x,y,z)=IFFT_{3D}{}^{\left(k_{x,}k_{y},k_{z}\right)}\left[Stolt^{\left(k\to k_{z}\right)}\left[S^{*}(k_{x},k_{y},k)\right]k_{z}e^{-jk_{z}z^{\prime }}\right]$$

where $Stolt^{\left(k\to k_{z}\right)}$ means the interpolation to resample the $k_{z}$ domain to uniformly spaced positions.

#### 2.2.2. Back Projection Algorithm

The gold-standard back-projection algorithm performs image reconstruction by coherently accumulating the signals of each transceiver antenna for each band in the time domain. This method can be applied to arbitrary array configurations, but with high computational complexity. Ignoring the loss path of the electromagnetic wave round trip, Equation (7) can be expressed as

(16) $$s(x^{\prime },y^{\prime },k)=∭f(x,y,z)e^{j2kR}dxdydz$$

where *R* are given in Equation (4). Equation (16) can be rephrased to recover the scattering coefficient function f(x,y,z) from the collected raw data $s(x^{\prime },y^{\prime },k)$ as [8]

(17) $$f(x,y,z)=∭s(x^{\prime },y^{\prime },k)e^{-2kR}dR$$

$s(x^{\prime },y^{\prime },k)$ is echo data and $e^{-2kR}$ is the calibration factor. The scattering coefficient function f(x,y,z) can be obtained by sequentially phase-calibrating the echo data in the time domain, traversing all orientation points. However, the traditional BPA has not considered the effect of clutter on imaging results under the non-ideal condition. The following sub-section provides an efficient solution for planar array imaging in non-ideal situation.

#### 2.2.3. Enhanced Back Projection Algorithm

In this section, compared to the traditional 3-D planar SAR imaging reconstruction methods using gold-standard BPA, this section proposes a beam-weighting-based Enhanced back-projection algorithm. Replace the amplitude factor and amplitude loss of $\frac{1}{R^{2}}$ in Equation (6) with the beam-weighting amplitude compensation, which can effectually reduce the adverse effects of the complex electromagnetic environment on image reconstruction and effectively suppress the ghost caused by antenna spacing greater than half-wavelength. The formula is derived as shown below.

Ignoring spatial noise, for an M-element spatial array with array element spacing $d(normally\;d=\frac{\lambda }{2})$, the array response vector for a uniform line array is expressed as [45]

(18) $$m\left(\theta \right)={\left[\begin{array}{llll} 1 & e^{\left(-j2\pi \frac{d}{\lambda }\sin\theta \right)} & \cdots & e^{\left(-j2\pi (M-1)\frac{d}{\lambda }\sin\theta \right)} \end{array}\right]}^{T}$$

where θ means the angle of azimuth of signal incidence and $\beta =\frac{2\pi d\sin\theta }{\lambda }$ The array output can be shown as

(19) $$Y=\sum_{l=1}^{M}e^{-j\frac{2\pi }{\lambda }(l-1)d\sin\theta }=\sum_{l=1}^{M}e^{-j(l-1)\beta }=\frac{\sin(\frac{M\beta }{2})}{M\sin(\frac{\beta }{2})}e^{j(M-l)\beta /2}$$

After normalization

(20) $$G=\frac{Y}{\left|Y\right|}=\left|\frac{\sin(M\beta /2)}{M\sin(\beta /2)}\right|$$

For the virtual array elements shown in Figure 1, each array element varies in beam direction from the image grid area. Here, we assume that the number of virtual array elements is N. Then, Equations (18) and (20) can be expressed as

(21) $$\begin{array}{l} m=\left[m_{1}\left(\theta \right),m_{2}\left(\theta \right),\cdots ,m_{N}\left(\theta \right)\right]\\ =\left[\begin{array}{cccc} 1 & 1 & \cdots & 1\\ e^{-j\frac{2\pi }{\lambda }d\sin\theta_{1}} & e^{-j\frac{2\pi }{\lambda }d\sin\theta_{2}} & \cdots & e^{-j\frac{2\pi }{\lambda }d\sin\theta_{N}}\\ \vdots & \vdots & \ddots & \vdots \\ e^{-j\frac{2\pi }{\lambda }(M-1)d\sin\theta_{1}} & e^{-j\frac{2\pi }{\lambda }(M-1)d\sin\theta_{2}} & \cdots & e^{-j\frac{2\pi }{\lambda }(M-1)d\sin\theta_{N}} \end{array}\right]\\ G=\left[G_{1},G_{2},\cdots ,G_{N}\right] \end{array}$$

Discretize $s(x^{\prime },y^{\prime },k)$ and the corrected phase of backscattered data correspond to the distance between the virtual array element and the target’s plane grid point. The critical step is to efficiently convert the coherent accumulation into a beam-weighting accumulation process and compensate for the signal amplitude of the imaging grid point. We can yield the 3-D scattering coefficient function $f(M_{i},M_{jk\prime },z)$. Hence, the Enhanced BPA image recovery process can be summarized as

(22) $$\;f(M_{i},M_{jk^{\prime }},z)=\left[\sum_{i=1}^{N}\sum_{j=1}^{J}\sum_{k^{\prime }=1}^{K}IFT^{\left(k_{z}\right)}\left[s(M_{i},M_{jk^{\prime }},k_{Stolt^{\left(k\to k_{z}\right)}})\right]e^{-j2kR_{M_{i},M_{jk^{\prime }}}}G_{M_{i},M_{jk^{\prime }}}W_{M_{i},M_{jk^{\prime }}}\right]$$

where $Stolt^{\left(k\to k_{z}\right)}$ denotes the Stolt interpolation, $IFT^{\left(k_{z}\right)}$ denotes 1-D inverse Fourier transform operation over the $k_{z}$ domain, and $M_{i}\;i=1,2,\cdot \cdot \cdot N$ means the virtual array elements, where N is the total number of virtual aperture. $M_{jk^{\prime }}$ represents the imaging grid matrix $j=1,2,\cdot \cdot \cdot J\;k^{\prime }=1,2,\cdot \cdot \cdot K$, where J,K are the imaging grid size. $W_{M_{i},M_{jk^{\prime }}}$ is the window function. The electromagnetic waves emitted by each virtual arrays are propagated into space as spherical waves. The latter is mapped to the imaging grid as a two-dimensional antenna pattern. Its amplitude compensation coefficient corresponds to the discrete normalized antenna beam.

The presented enhanced method is similar to the gold standard BPA. It improves the quality of the image using beam-weighting to achieve amplitude compensation in the non-ideal electromagnetic environment. This approach delivers high-fidelity image reconstruction focusing on coherently accumulating the received signal from each transceiver pair. Although its computational complexity is enormous [12,32,43], we can use the integrated parfor-function or GPU of MATLAB to speed up the computation.

### 2.3. YOLO Detection Network

**Figure 2:**
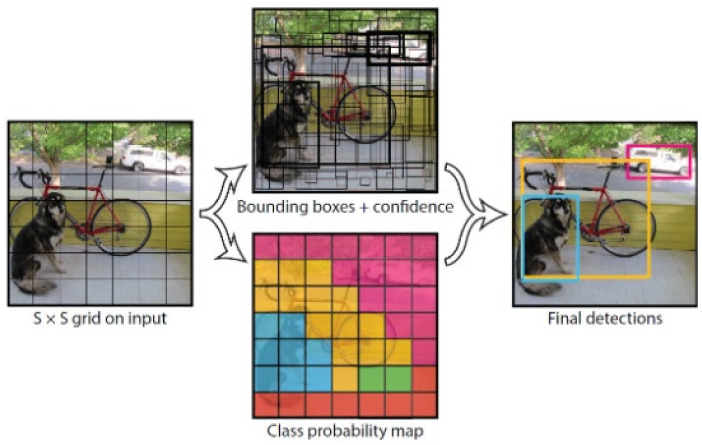
The system of detection models.

YOLO (you only look once) provides a fast detection and high accuracy method for target detection, which takes the target detection problem as a predictive regression problem of target regions and categories. In this method, a single neural network has been used to directly predict the probabilities of target regions and categories, providing end-to-end target detection. Compared to conventional target detection, it has the characteristics of simpler process, faster speed, and easier training. YOLO unifies the target recognition process into a neural network to predict the bounding box category of the target by utilizing the complete image information. This network structure has been investigated in the literature and employed as a detection method in this paper[44]. The model is shown in Figure 2.

Class probability includes the probability of the prediction frame containing the target and the accuracy of the prediction frame. It is assumed that the YOLO algorithm detects the targets of n categories, then the class probability of the detected targets in this cell belonging to n categories can be expressed as

(23) $$P_{r}(class_{i}\left|object)*\right.P_{r}(object)*IOU_{pred}^{truth}=P_{r}(class_{i})*IOU_{pred}^{truth}$$

where $P_{r}(class_{i})*IOU_{pred}^{truth}$ is the confidence level and $IOU_{pred}^{truth}$ is the ratio of the intersection between the prediction box and the actual box.

## 3. Imaging Results and Evaluation

**Figure 3:**
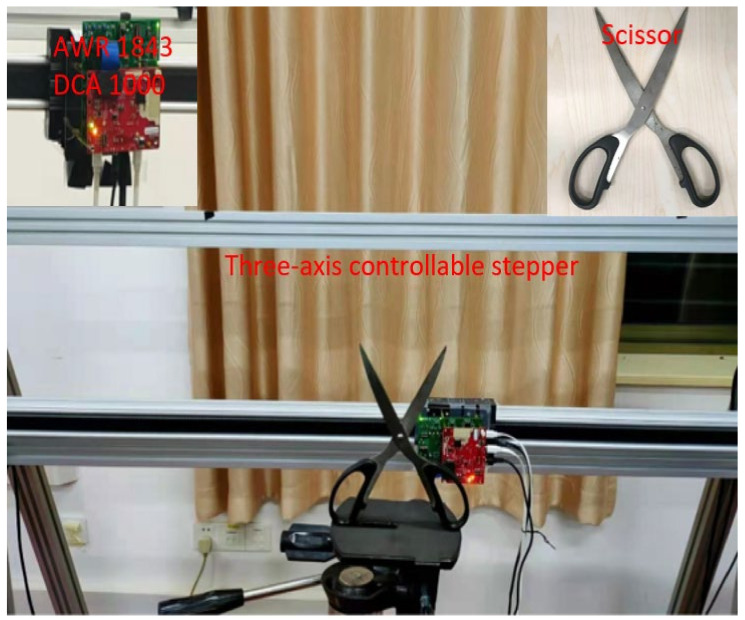
The experimental equipment of SAR system.

The parameter configuration of the experimental platform is described, and the results of imaging are used to evaluate these three imaging algorithms. As shown in Figure 3, the system devices include a three-axis controllable stepper, a 77G ‘AWR1843’ mmWave radar sensor, which can generate 77–81 GHz linear frequency modulated continuous waves, and a ‘DCA1000’ high-performance raw data acquisition card by Texas Instruments (TI), personal computer (PC), and the target.

PC, as the control center, connects with the mmWave radar sensor “AWR1843”, high-performance raw data acquisition card “DCA1000”, and the three-axis controllable stepper. When the mmWave radar and three-axis controllable stepper are in simultaneous operation, the “AWR1843” receives the echo signals on each sub-aperture point by point. It collects the raw data at high speed by DCA1000 and subsequently transfers the echo data to PC via the network.

In this paper, the above algorithm scheme is validated for mmWave FMCW-SAR accurate measurements. The platform works by scanning through a Z−trajectory, and the mmWave radar images the scissor and wrench in forward and side-looking modes. The radar system parameters are shown in Table 1.

**Figure 4:**
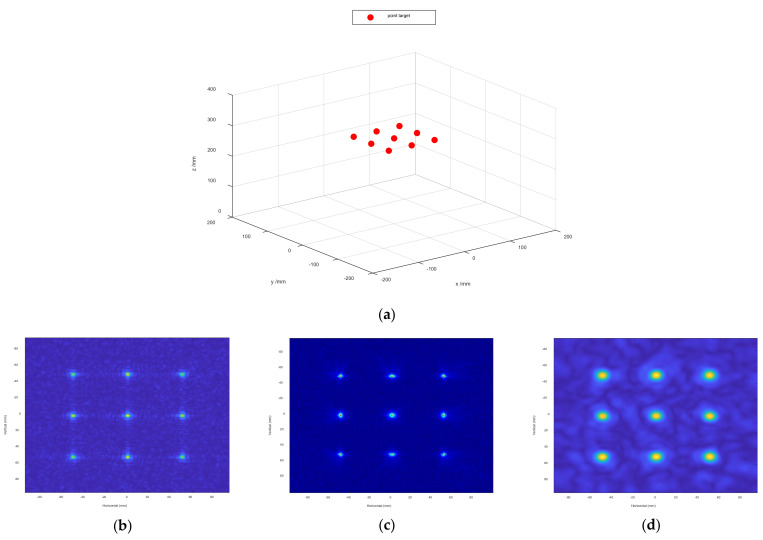
Spatial target model of multiple points in a 60 dBW gaussian white noise environment. (**a**) are the multiple points. (**b**) is the 2-D multiple points imaging result in BPA. (**c**) is the 2-D multiple points imaging result in RMA with amplitude loss compensation. (**d**) is the 2-D multiple points imaging result in Enhanced BPA.

In order to verify the superiority of the proposed algorithm in a 60 dBW gaussian white noise environment, we perform a simulation using the radar parameters of the scissor group described in the previous section to validate it. As shown in Figure 4a, the spatial targets model with nine points are distributed in a non-ideal space. Figure 4b–d show the 2-D SAR image. Compared with the image result based on these algorithms, the proposed algorithm can improve the measurement accuracy of RCS.

**Figure 5:**
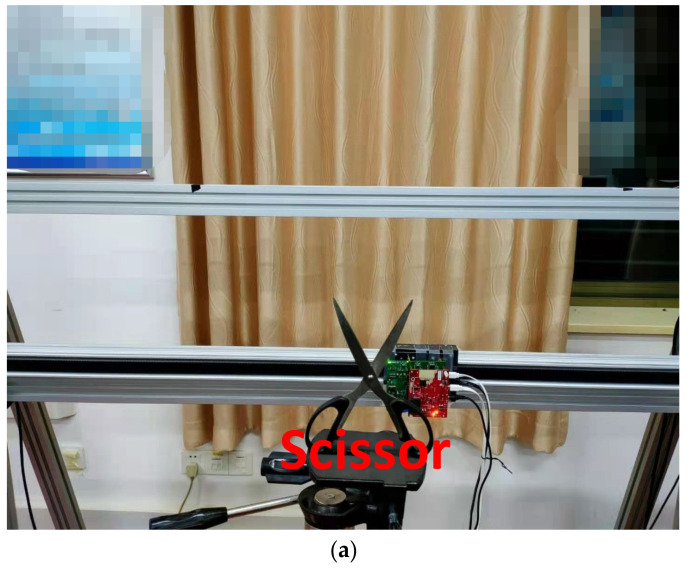

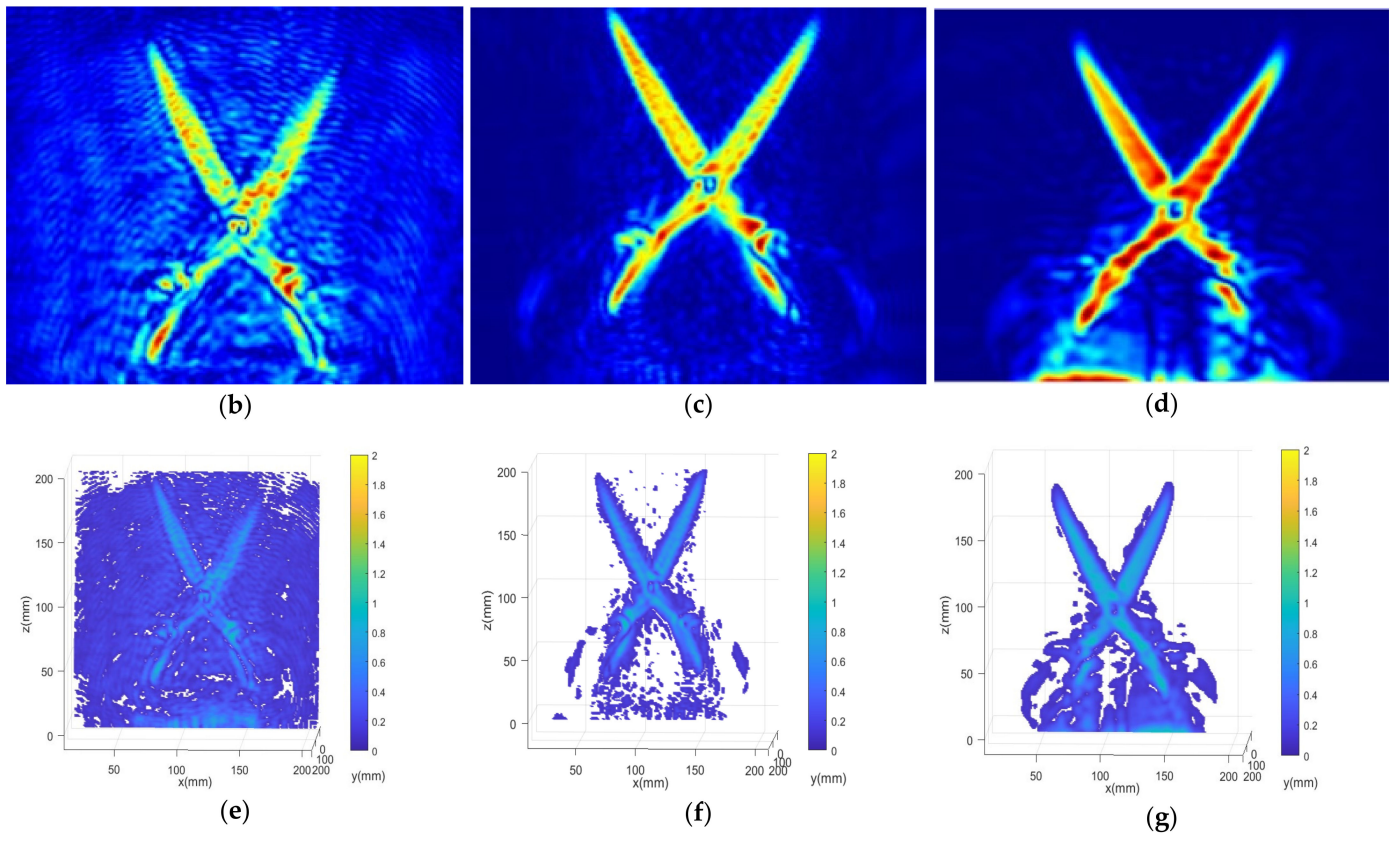
Imaging results in a non-ideal environment. (**a**) is the scissor optical image. (**b**) is the 2-D scissor imaging result in BPA. (**c**) is the 2-D scissor imaging result in RMA with amplitude loss compensation. (**d**) is the 2-D scissor imaging result in Enhanced BPA. (**e**) is the 3-D scissor imaging result in BPA. (**f**) is the 3-D scissor imaging result in RMA with amplitude loss compensation. (**g**) is the 3-D scissor imaging result in Enhanced BPA.

Figure 5 presents results of the scissor image reconstruction based on the three imaging algorithms. The scissor group is surrounded by angular reflection and scatterers, and the distance between the reference planar and the target is $z_{0}$ = 280 mm. The number of space sampling locations is chosen in an approximate virtual array from −100 mm to 100 mm along the x and y directions. To satisfy the sampling condition, the distance between adjacent sampling points is usually less than λ/4. Here, the horizontal and vertical space sampling intervals are set as 0.5 mm and 1 mm, respectively. Figure 5a shows the optical image of the experimental scissor model. Figure 5b–d show the 2-D SAR image. After the basic image filtering, 3-D SAR image under these three algorithms are obtained, as seen in Figure 5e–g. A comparison between Figure 5b,d indicates the scissor handle is completely submerged in the side lobes clutter under the BPA. Furthermore, severe loss of detail and edge diffraction can be observed. The scissors in the image under the RMA with amplitude loss compensation are well imaged, only with some streaks on their object contours. However, the side lobes clutter of the image has effectively been suppressed, with the most remarkable focusing result under the enhanced BPA.

**Figure 6:**
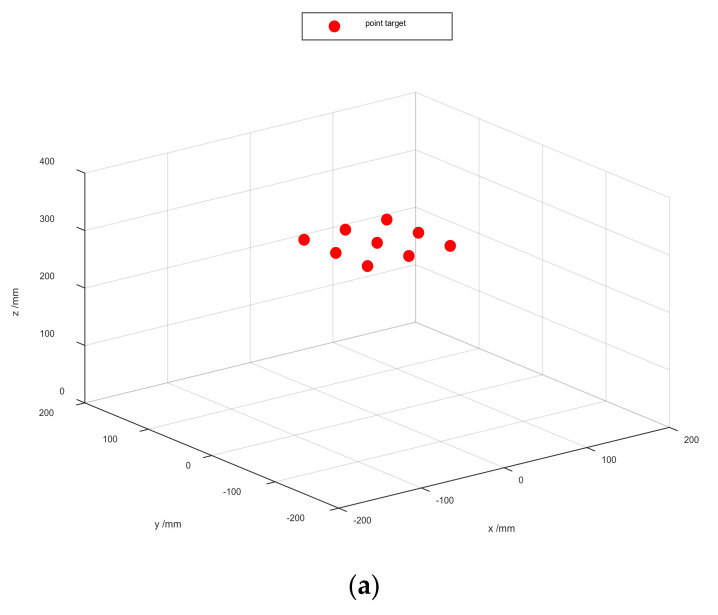

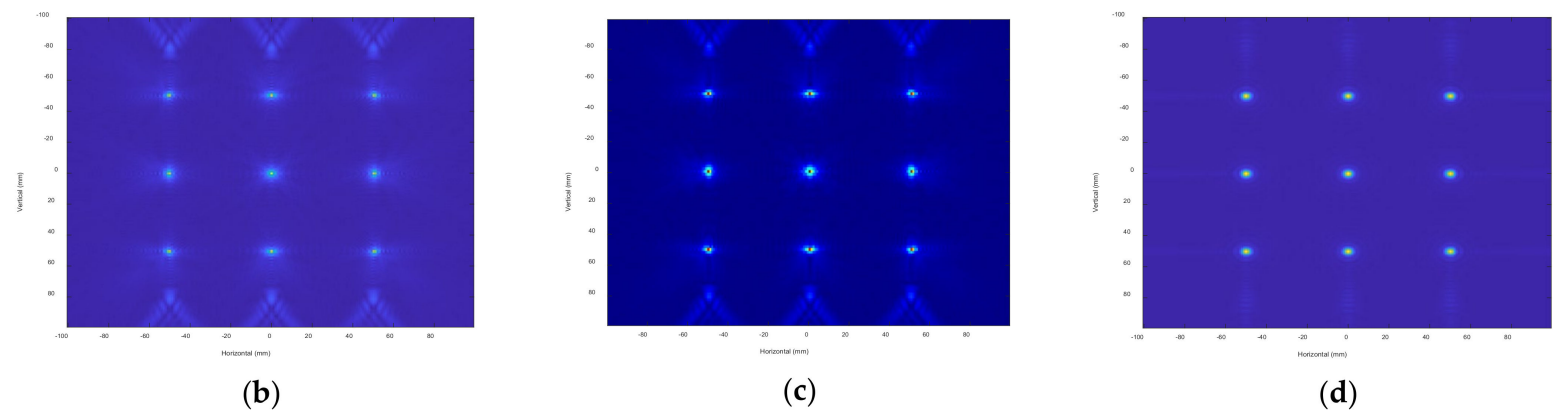
Spatial target model of multiple points in an ideal space. (**a**) are the multiple points. (**b**) is the 2-D multiple points imaging result in BPA. (**c**) is the 2-D multiple points imaging result in RMA with amplitude loss compensation. (**d**) is the 2-D multiple points imaging result in Enhanced BPA.

Figure 6 shows the simulation using the radar parameters of the wrench group. As shown in Figure 6a, the spatial targets model with nine points are distributed in an ideal space. Figure 6b–d show the 2-D SAR image. When the vertical spacing is greater than half a wavelength, we can clearly observe that the proposed algorithm can suppress the ghost more effectively than the others.

**Figure 7:**
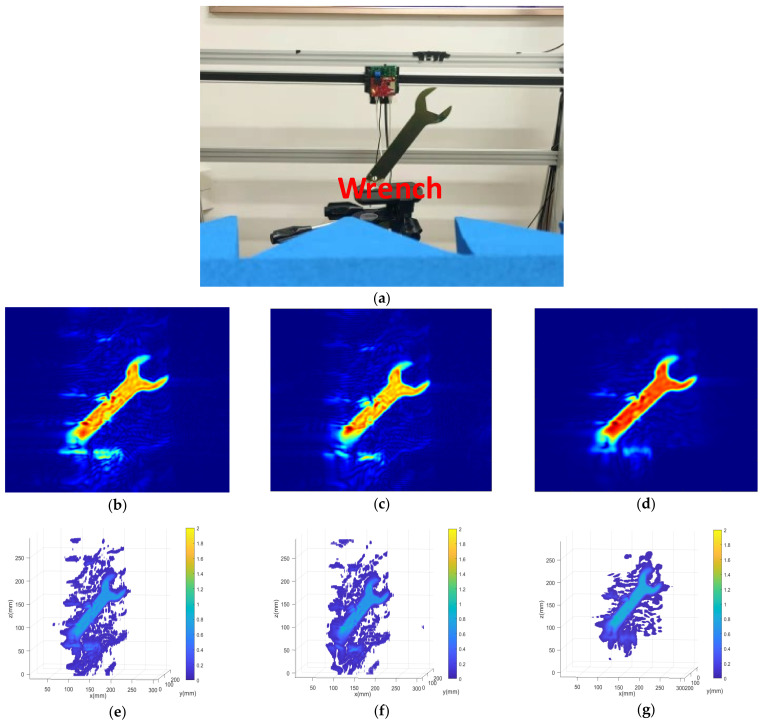
Imaging results in dark environment. (**a**) is the wrench optical image. (**b**) is the 2-D wrench imaging result in BPA. (**c**) is the 2-D wrench imaging result in RMA with amplitude loss compensation. (**d**) is the 2-D wrench imaging result in Enhanced BPA. (**e**) is the 3-D wrench imaging result in BPA. (**f**) is the 3-D wrench imaging result in RMA with amplitude loss compensation. (**g**) is the 3-D wrench imaging result in Enhanced BPA.

The image reconstruction results of the wrench are shown in Figure 7. The wrench group is placed in the darkroom environment and the distance between the reference planar and the target is $z_{0}$ = 300 mm. The number of space sampling locations is chosen in an approximate virtual array from −150 mm to 150 mm along the x and y directions. The horizontal space sampling interval is selected as 0.5 mm. The vertical distance of antenna space sampling intervals is chosen to be 2 mm greater than half a wavelength, leading to the ghost image. Figure 7a shows the optical image of the experimental wrench model. Figure 7b–d show the 2-D SAR image. Figure 7e–g show the 3-D SAR image via these three algorithms. Notice that the images under the BPA and the RMA with amplitude loss compensation have more ghosts and streaks on the object contour. In contrast, the image reconstruction under the enhanced BPA is optimal, in which the ghost image can be effectively suppressed.

In fact, BPA and RMA are essentially the same imaging algorithms, while the former starts from the time domain and the latter from the frequency domain. In the time domain, the energy of each imaging grid point is distributed over all synthetic apertures. BPA compensates for the data of all synthetic aperture points on the imaging grid. At the same time, RMA uses the Fourier transform to fix the energy of each target scattering point at a frequency point in the wavenumber domain, which enables point-to-point focus.

However, in the real scenario, under near-field conditions, where the electromagnetic wave is spherical, the data of the distance cell between the virtual point and imaging grid point contain the scattered energy of the target and other unrelated targets at the same distance. At the same time, the near-field condition means each radar beam could not fully illuminate the target. The traditional BPA will gather other clutter energy at the same distance, thus affecting the image quality. In addition, the sparse space sampling in the virtual array also causes the ghost image due to the appearance of grating lobes on the frequency spectrum.

As for the solution to address side lobes clutter and the ghost, the proposed algorithm sets a beam-weighting method based on the antenna orientation map. The amplitude compensation is applied to the energy of each virtual point with all imaging grid points, and then all virtual points are traversed. From the perspective of the frequency spectrum, the side lobes and grating lobes are suppressed. In essence, the enhanced BPA is designed to make the data on the imaging grid points as much as possible to achieve the real target RCS.

To further evaluate the image quality under these three algorithms, quantitative analysis of the images, azimuth and height directional magnitude profiles, image contrast, and entropy are introduced in the later sections.

**Figure 8:**
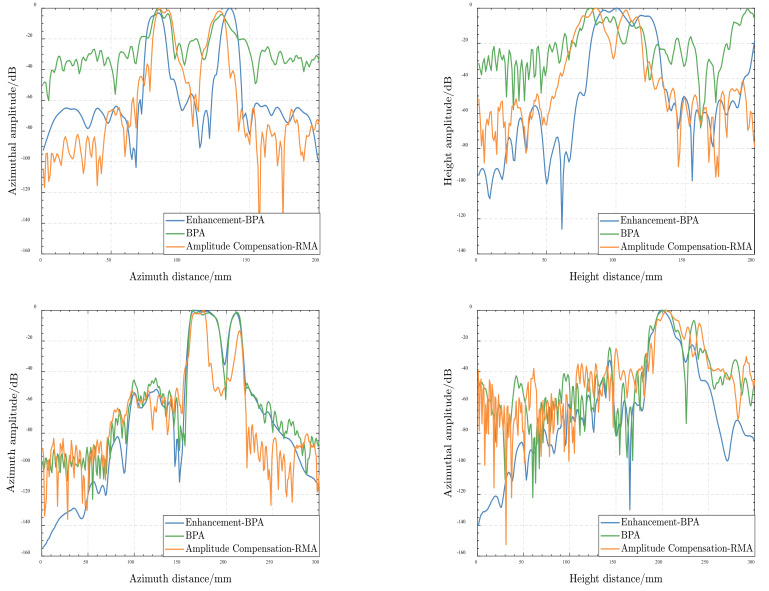
Azimuthal and height profiles. Row 1 is the azimuthal and height profiles of the scissor, while row 2 is the azimuthal and altitudinal profile of the wrench. Column 1 is the Azimuth profiles of the targets; Column 2 is the height profiles of the targets.

In order to verify the imaging performance of the proposed algorithm, Figure 8 shows the azimuth and height direction amplitude image of the scissor and wrench, respectively, for these three algorithms. Row 1 is the azimuthal and height profiles of the scissor. The azimuthal direction shows that the enhanced BPA reduces the intensity of the side lobes clutter by 30–40 dB compared with the BPA. Comparing the RMA with amplitude loss compensation in the scissor, the azimuthal image of the two principal lobes is narrower in the proposed algorithm, which also indicates a higher focusing performance. Row 2 represents the azimuthal and height profiles of the wrench. In the azimuthal direction, the proposed algorithm simply degrades the side lobes compared to the gold-standard BPA. It achieves the same imaging performance as the RMA with amplitude loss compensation. In the height direction, it can be clearly seen that the proposed algorithm shows a drop of at least 20 dB in grating lobes. Therefore, the proposed method boasts the better image, higher fidelity focusing ability, and effectively suppresses the ghost image under non-ideal electromagnetic cases or sparse arrays, compared with the others.

As shown in Figure 8, when the scenario environment is non-ideal or arrays are sparse, there are more grating lobes or side lobes clutter on the image using the traditional BPA and RMA with amplitude loss compensation, contributing to worse image quality. To further evaluate the image quality, the image contrast and entropy are introduced [45] here. The image contrast is the difference of color in an image, indicating the image texture characteristics. The higher the contrast, the more visible the image details. Entropy represents the degree of system disorder. The image entropy can indicate the quality of image focusing. The smaller the entropy, the better the effectiveness of focusing. The image contrast and entropy can be expressed as

(24) $$I_{Contrast}=\sqrt{\frac{MN}{{\left({\left\|\alpha_{ij}\right\|}_{2}\right)}^{2}}\sum_{i=1}^{M}\sum_{j=1}^{N}{\left|\alpha_{ij}\right|}^{4}}\;I_{Entropy}=\sum_{i=1}^{M}\sum_{j=1}^{N}\frac{{\left|\alpha_{ij}\right|}^{2}}{{\left({\left\|\alpha_{ij}\right\|}_{2}\right)}^{2}}\log\frac{{\left|\alpha_{ij}\right|}^{2}}{{\left({\left\|\alpha_{ij}\right\|}_{2}\right)}^{2}}$$

For imaging under three different algorithms, the values of the image contrast and entropy are also rational, as shown in Table 2, From the viewpoint of the image quality. the enhanced BPA contrast is larger than the others, and the image entropy is lower. It means that the texture detail and image quality recovery is improved with the proposed method in radar imaging.

## 4. Discussion

**Figure 9:**
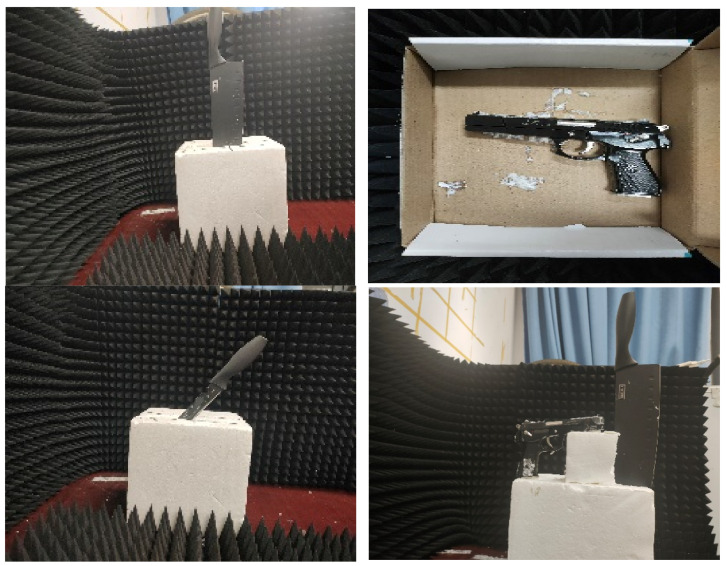
Optical image in different targets.

**Figure 10:**
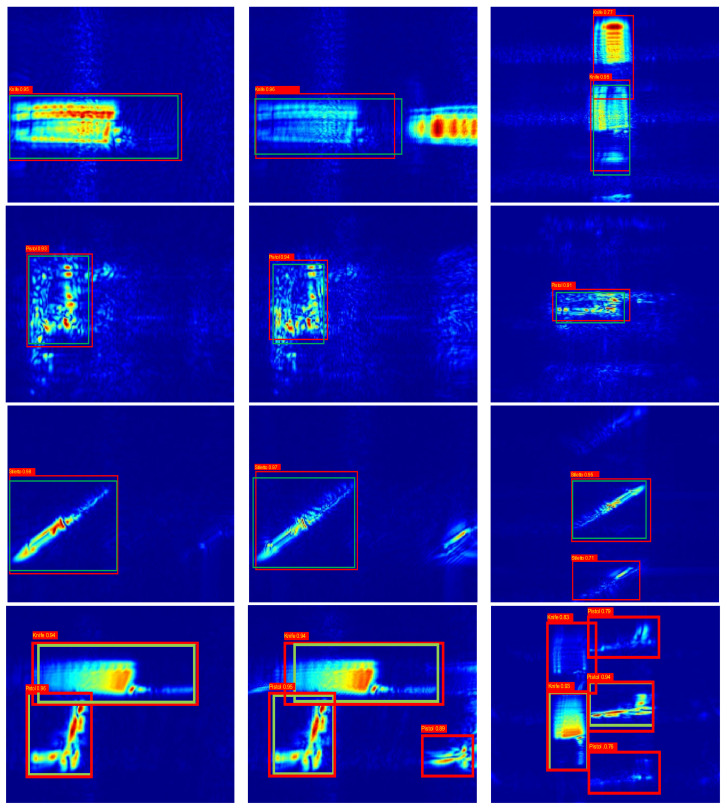
Detection and identification profiles. Row 1 is the knife of SAR images under the three imaging algorithms. Row 2 is the concealed pistol of SAR images under the three imaging algorithms. Row 3 is the stiletto of SAR images under the three imaging algorithms. Row 4 is the pistol and knife of SAR images under the three imaging algorithms. Column 1 is the Enhanced BPA imaging detection results. Column 2 is the BPA imaging detection results. Column 3 is the RMA with amplitude loss compensation imaging results.

To further validate the strong robustness of the proposed algorithms in complex environments, 3DRIED was used to construct the image datasets of these three imaging algorithms, and the YOLO algorithm was used for detection and evaluation. Figure 9 shows the different targets of optical images, such as knife, concealed pistol, stiletto and multi-target. Figure 10 shows the detection rates for different targets, with the green and red boxes being the true and prediction boxes, respectively. The prediction box has the category and confidence level in the upper left corner. It can be observed that YOLO can effectively detect the imaged objects. Since the radar antenna spacing in 3DRIED is larger than half a wavelength, the SAR images based on conventional algorithms are affected by the ghost. Due to the presence of the ghost image, the neural network often misidentifies shadows as targets, which affects the accuracy of detection.

The kappa coefficient is introduced further to measure the image evaluation accuracy [46]. It is used to evaluate the accuracy of the classification model. The higher the coefficient, the better the classification accuracy of the image. The range of this coefficient is [−1,1], and in practice, it is usually [0, 1]. The kappa coefficient can be expressed as

(25) $$\kappa =\frac{P_{accuracy}-P_{expected\;accuracy}}{1-P_{expected\;accuracy}}$$

where $P_{accuracy}$ is the accuracy of the samples, and $P_{expected\;accuracy}$ is the expected accuracy of the samples. The kappa coefficient of images classification are shown in Table 3. It is evident that the proposed algorithm could efficiently suppress the ghost image, and its kappa coefficient is improved compared to the other two algorithms.

## 5. Conclusions

In this paper, we design a 3-D near-field mmWave SAR imaging platform and propose an image focusing algorithm. Our technique extends the traditional BPA by introducing beam-weighting. The novel algorithm is proposed to efficiently suppress grating lobes or side lobes clutter on the image, applicable to a diverse set of complex environments. By conducting data acquisition, we analyze and evaluate the parameters of the imaging results using these algorithms. The results demonstrate the robustness of our approach in the presence of interference from external factors. Furthermore, we use the 3DRIED to construct image datasets to validate the proposed algorithm. Our algorithm achieves high-fidelity image reconstruction, focusing on experimental studies and detection results, which provides high-quality images as input for subsequent applications. The detection recognition rate is higher. Our purpose is to contribute a complete and efficient imaging and detection identification process for facilitating industrial research.

## Figures and Tables

**Table 1:** The parameters of the SAR platform.

Parameter Type	Numerical Value	Unit
Centre Carrier Frequency	79	GHz
Platform Speed	20	mm/s
Pulse Repetition Period	25	ms
Bandwidth	4	GHz
Range Resolution	3.75	cm
Azimuth resolution	0.5	mm
Height resolution	0.5	mm
Azimuth to Sub-aperture spacing	0.5	mm
Scissor Height to sub-aperture spacing	1	mm
Wrench Height to sub-aperture spacing	2	mm
Scissor Synthetic Aperture Size	200 × 200	mm^2^
Wrench Synthetic Aperture Size	300 × 300	mm^2^
Vertical Distance between Scissor and Radar	280	mm
Vertical Distance between Wrench and Radar	300	mm

**Table 2:** Quality evaluation of image contrast and entropy in different environments.

Evaluation Systems	Enhanced BPA	BPA	Amplitude Compensation-RMA
$I_{Contrast}^{Scissor}$	134.5673	89.5611	112.6986
$I_{Entropy}^{Scissor}$	3.8054	4.2688	3.8837
$I_{Contrast}^{Wrench}$	220.877	188.3848	191.0390
$I_{Entropy}^{Wrench}$	3.7456	3.8230	3.8499

**Table 3:** Quality evaluation of kappa coefficient in different objects.

Kappa Coefficients	Enhancement-BPA	BPA	Amplitude Compensation-RMA
$\kappa_{knife}$	0.92	0.87	0.85
$\kappa_{pistol}$	0.93	0.92	0.91
$\kappa_{stiletto}$	0.95	0.86	0.89
$\kappa_{pistol,knife}$	0.93	0.87	0.81

## Data Availability

https://github.com/zzzc1n/3-D-HPRID.git (accessed on 1 March 2022).
